# Biphasic transcriptional and posttranscriptional regulation of MYB by androgen signaling mediates its growth control in prostate cancer

**DOI:** 10.1016/j.jbc.2022.102725

**Published:** 2022-11-19

**Authors:** Srijan Acharya, Shashi Anand, Mohammad Aslam Khan, Haseeb Zubair, Sanjeev Kumar Srivastava, Seema Singh, Ajay Pratap Singh

**Affiliations:** 1 Department of Pathology, College of Medicine, University of South Alabama, Mobile, Alabama, USA; 2 Cancer Biology Program, Mitchell Cancer Institute, University of South Alabama, Mobile, Alabama, USA; 3 Department of Biochemistry and Molecular Biology, College of Medicine, University of South Alabama, Mobile, Alabama, USA

**Keywords:** prostate cancer, androgen receptor, oncogene, MYB, DHT, transcription promoter, MicroRNA mechanism, MicroRNA-150, gene regulation, AR, androgen receptor, ARE, androgen-responsive element, ChIP, chromatin immunoprecipitation, CSS, charcoal-stripped serum, DHT, dihydrotestosterone, HRP, horseradish peroxidase, PCa, prostate cancer, qRT-PCR, quantitative RT-PCR, seAP, secreted alkaline phosphatase

## Abstract

*MYB*, a proto-oncogene, is overexpressed in prostate cancer (PCa) and promotes its growth, aggressiveness, and resistance to androgen-deprivation therapy. Here, we examined the effect of androgen signaling on *MYB* expression and delineated the underlying molecular mechanisms. Paralleling a dichotomous effect on growth, low-dose androgen induced *MYB* expression at both transcript and protein levels, whereas it was suppressed in high-dose androgen-treated PCa cells. Interestingly, treatment with both low- and high-dose androgen transcriptionally upregulated *MYB* by increasing the binding of androgen receptor to the *MYB* promoter. In a time-course assay, androgen induced *MYB* expression at early time points followed by a sharp decline in high-dose androgen-treated cells due to decreased stability of *MYB* mRNA. Additionally, profiling of *MYB*-targeted miRNAs demonstrated significant induction of miR-150 in high-dose androgen-treated PCa cells. We observed a differential binding of androgen receptor on miR-150 promoter with significantly greater occupancy recorded in high-dose androgen-treated cells than those treated with low-dose androgen. Functional inhibition of miR-150 relieved *MYB* suppression by high-dose androgen, while miR-150 mimic abolished *MYB* induction by low-dose androgen. Furthermore, *MYB*-silencing or miR-150 mimic transfection suppressed PCa cell growth induced by low-dose androgen, whereas miR-150 inhibition rescued PCa cells from growth repression by high-dose androgen. Similarly, we observed that *MYB* silencing suppressed the expression of androgen-responsive, cell cycle–related genes in low-dose androgen-treated cells, while miR-150 inhibition increased their expression in cells treated with high-dose androgen. Overall, these findings reveal novel androgen-mediated mechanisms of *MYB* regulation that support its biphasic growth control in PCa cells.

Prostate cancer (PCa) is the most frequently diagnosed malignancy affecting millions of men worldwide (1, 2). It is also the second leading cause of cancer-related death in American men (3) Androgen signaling plays a central role in the pathogenesis of PCa development, and patients with locally advanced or metastatic PCa are treated with androgen-deprivation or castration therapy (4, 5). However, despite an initial response, PCa relapses in most patients in a castration-resistant (CR) form, which is more aggressive. CR PCa responds to alternative therapies that inhibit androgen synthesis or interfere with its binding to the androgen receptor (AR), but these therapies also fail, eventually contributing to the patient’s mortality (5, 6, 7). Interestingly, a dichotomous response of AR signaling is also well documented where it promotes PCa growth at lower androgen levels while suppressing it at high doses of androgens (8, 9, 10). However, the precise mechanisms underlying this dose-dependent biphasic growth response are not yet well understood.

*MYB* is a cellular counterpart of the *v-myb* oncogene carried by the chicken retroviruses AMV and E26 that cause acute myeloblastic leukemia or erythroblastosis (11, 12). It encodes for a transcription factor protein, which activates gene expression in most cases upon binding to the responsive promoter elements (13). *MYB* was earlier thought to have a restricted expression in the immature hematopoietic cells, which decreased as the cells differentiated into different lineages (14). Later, *MYB* expression was detected in other tissues, especially in the stem and progenitor cells of the colon and breast, as well as hematological and some solid malignancies (15, 16, 17, 18, 19). *MYB* amplification has been reported in PCa, and its amplification frequency increases in hormone-refractory disease (20). We and others have shown that MYB plays a significant role in PCa growth, aggressive malignant phenotypes, and castration resistance (17, 21, 22). Interestingly, while we observed a cooperative role of MYB in supporting ligand-independent AR activity, another study suggested its compensatory action demonstrating its transcriptional upregulation following impairment of the AR signaling (21).

In this study, we examined the role of androgen signaling in *MYB* expression in PCa cells and delineated the underlying regulatory mechanisms and functional significance. We report that MYB has no significant effect on *AR* expression; however, androgen signaling regulates *MYB* expression in a dose-dependent biphasic manner paralleling its effect on the growth of PCa cells. Interestingly, androgen, at both low and high doses, induced *MYB* expression at the transcriptional level, and *MYB* repression by high-dose androgen occurred late, involving a miR-150–mediated posttranscriptional mechanism. Inhibition of miR-150 abrogated the repressive effect of high-dose androgen on *MYB* expression and the growth of PCa cells. Moreover, MYB silencing also attenuated the growth-promoting effect of androgen on PCa cells, suggesting their cooperative role in cancer pathogenesis. Collectively, these data provide a novel insight into the dichotomous growth response of AR signaling in PCa.

## Results

### Androgen signaling regulates MYB expression in PCa cells in a dose-dependent biphasic manner

**Figure 1:**
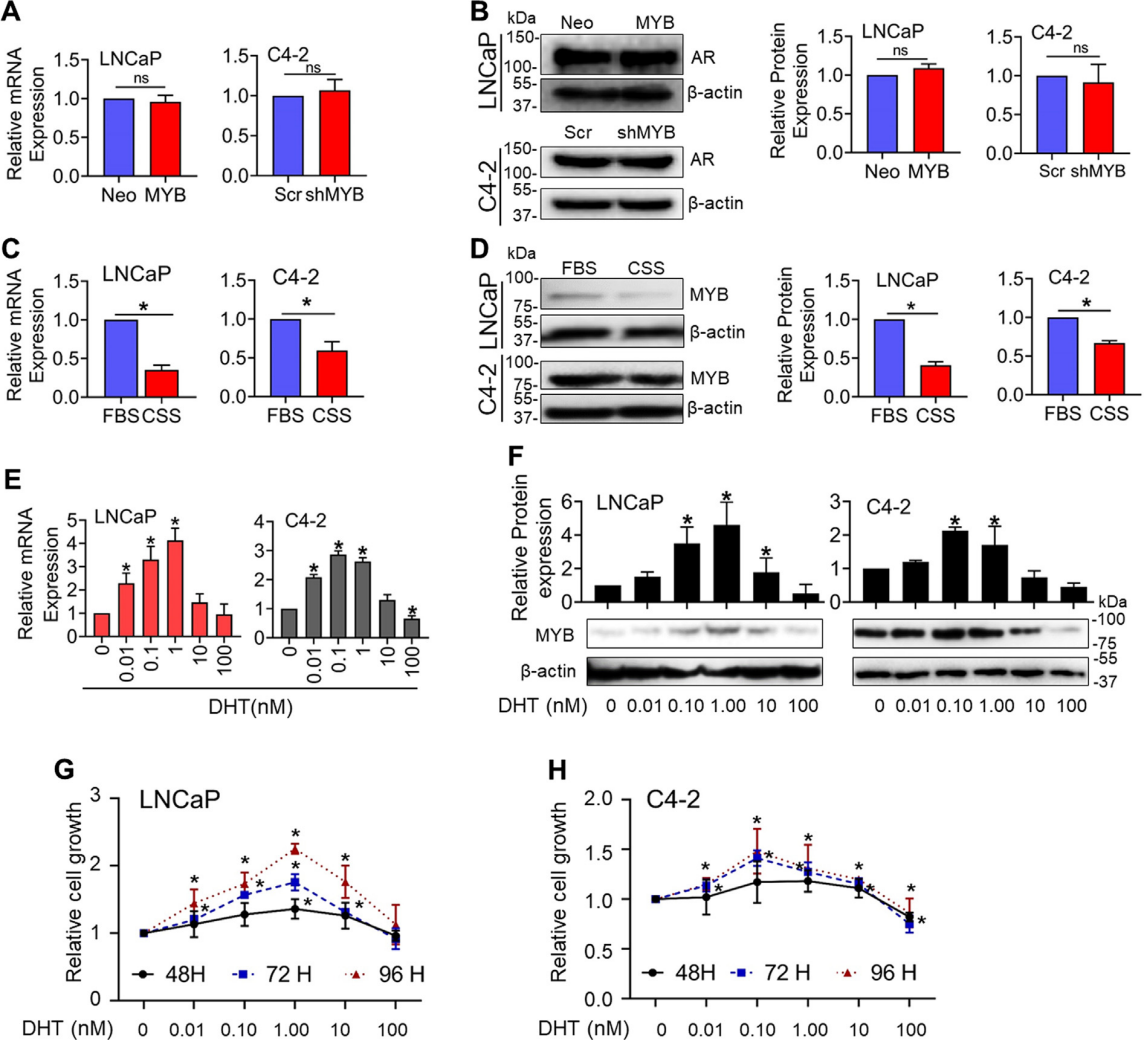
**Androgen regulates MYB expression in a dose-dependent biphasic manner.** Low (LNCaP-Neo/C4-2-shMYB) and high (LNCaP-MYB/C4-2-Scr) MYB-expressing prostate cancer cells were cultured in media supplemented with FBS, and AR expression was examined at transcripts (*A*) and protein (*B*) levels using qRT-PCR and immunoblotting, respectively. *ACTB* (for qRT-PCR) and β-actin (for immunoblot assay) were used as internal controls. Protein band intensity was quantified by densitometry, and the data were plotted as a bar graph. LNCaP and C4-2 cells were grown in a regular medium (supplemented with FBS) to subconfluence. Subsequently, the medium was replaced with fresh FBS- or charcoal-stripped serum (CSS)–containing medium. After 48 h of incubation, MYB expression was analyzed at both mRNA (*C*) and protein (*D*) levels. For a dose-response, cells were treated with increasing concentrations of DHT (0–100 nM) for 48 h in CSS-containing media, and MYB expression was examined at mRNA (*E*) and protein (*F*) levels. *G* and *H*, cells were treated with DHT (0–100 nM) in CSS media and the change in growth measured at different time intervals (48–96 h). The data is presented as line graphs. ∗ indicates statistical significance (*p* < 0.05), n.s. not significant. AR, androgen receptor; DHT, dihydrotestosterone.

To investigate the effect of MYB modulation on *AR* expression, we used C4-2 and LNCaP cells that had been engineered for either *MYB* silencing (C4-2-shMYB) or forced *MYB* overexpression (LNCaP-MYB) (Fig. S1). Thereafter, we examined the expression of AR in these cell lines. No significant changes in AR expression at both mRNA and protein levels were observed (Fig. 1, *A* and *B*). Next, we cultured LNCaP and C4-2 PCa cells in steroid-depleted (charcoal-stripped serum, CSS) and steroid-supplemented (FBS) media for 48 h to observe if androgen depletion had an effect on *MYB* expression. Our results show that the expression of *MYB* was reduced significantly at both mRNA and protein levels in cells cultured in androgen-depleted media compared to those grown in androgen-supplemented condition (Fig. 1, *C* and *D*). In additional experiments, we silenced the expression of *AR* in LNCaP and C4-2 cells by RNA interference and examined its effect on *MYB* expression. We observed that the expression of MYB was reduced significantly at both protein and mRNA levels in AR-silenced LNCaP and C4-2 cells (Fig. S2, *A* and *B*). To further confirm the role of androgen signaling in *MYB* regulation, we treated LNCaP and C4-2 cells with increasing doses (0–100 nM) of dihydrotestosterone (DHT). We observed that DHT treatment induced MYB expression at lower doses, followed by a decrease at high doses at both RNA and protein levels (Fig. 1, *E* and *F*). In LNCaP cells, MYB expression increased at treatment doses of up to 1.0 nM, whereas a slight decline in induced MYB expression was noted at 1.0 nM in C4-2 cells. Interestingly, this biphasic regulation of MYB paralleled the biphasic effect of DHT treatment on the growth of LNCaP and C4-2 cells (Fig. 1, *G* and *H*). This biphasic effect of DHT on MYB and growth was further confirmed in two additional AR-expressing PCa cell lines, LAPC-4 and VCaP (Fig. S3). These findings suggest that MYB and AR engage in regulatory crosstalk in PCa cells with potential significance in growth response.

### Androgen treatment induces the transcriptional activity of the *MYB* promoter and enhances the binding of AR to the responsive elements

**Figure 2:**
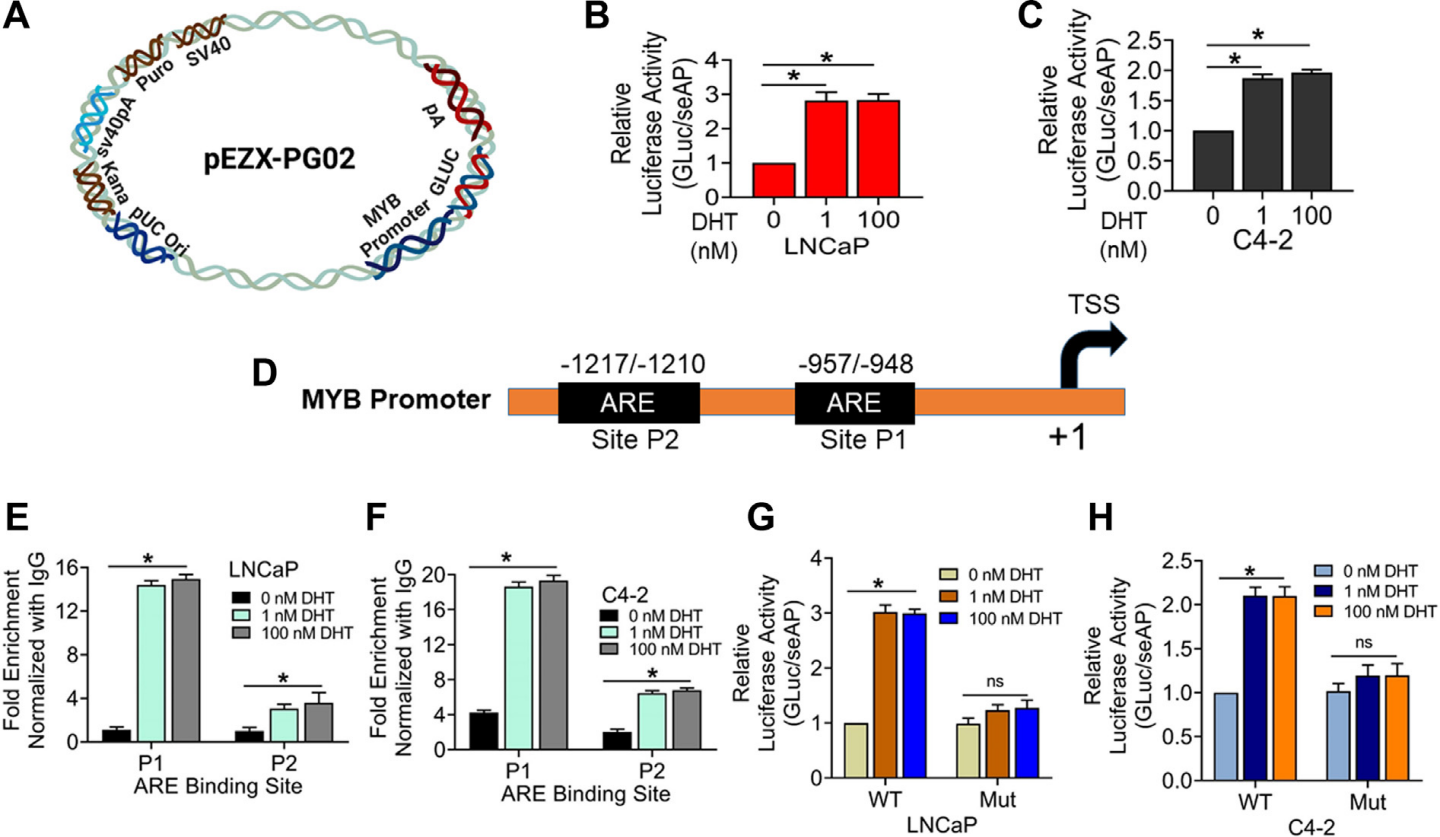
**Androgen treatment induces the transcriptional activity of MYB promoter at both low and high doses in prostate cancer cells.***A*, a reporter plasmid containing *MYB* promoter sequence upstream of the luciferase gene. *B* and *C*, LNCaP and C4-2 cells were cotransfected with the promoter-reporter plasmid and secreted-alkaline phosphatase (seAP) gene downstream of CMV promoter to control for transfection efficiency. After 24 h, cells were treated with either low (1 nM) or high (100 nM) DHT. Cells were lysed after 24 h and luciferase activity was measured and presented after normalization with seAP activity (mean ± S.D.), ∗*p* = < 0.05. *D*, *in silico* analysis of the *MYB* promoter identified two androgen-responsive elements (AREs) in the *MYB* promoter. *E* and *F*, LNCaP and C4-2 cells treated with low or high doses of DHT for 24 h were subjected to chromatin immunoprecipitation using anti-AR antibody or control IgG, followed by qPCR using primers flanking AREs. Fold enrichment (mean ± S.D.) was calculated from three replicates. *G* and *H*, LNCaP and C4-2 cells were transfected with WT or P1 site–mutated (Mut) *MYB* promoter-reporter plasmids along with control plasmid (seAP) for 24 h and thereafter treated with either low (1 nM) or high (100 nM) DHT doses. After 24 h of treatment, luciferase activity was measured and presented after normalization with seAP activity (mean ± S.D.), ∗*p* = < 0.05, n.s. not significant. AR, androgen receptor; DHT, dihydrotestosterone.

Since we observed an increase in MYB expression following DHT treatment at both mRNA and protein levels, we examined the possibility of its transcriptional regulation. PCa cells were cotransfected with MYB promoter-driven Gaussia luciferase reporter plasmid and secreted alkaline phosphatase (seAP) under the control of constitutive CMV promoter construct to control the transfection efficiency (Fig. 2*A*). After 24 h of transfection, cells were treated with an MYB-inducing low (1.0 nM) and a MYB-repressive high dose (100 nM) of DHT, and the changes in the luciferase activity were measured after 24 h. The data show that luciferase activity was increased by ∼ 2.7-fold and ∼ 1.9-fold in LNCaP and C4-2 cells, respectively, treated with both low and high doses of DHT (Fig. 2, *B* and *C*). Observing induction of *MYB* promoter transcriptional activity upon DHT treatment, we surveyed *MYB* promoter for putative androgen-responsive elements (AREs) by *in silico* analysis using PROMO-ALGGEN application. Two putative AREs were identified upstream of the *MYB* transcription start site and named P1 (-948/-957) and P2 (-1210/-1217) by using PROMO-ALGGEN and TFBIND (Fig. 2*D*). Thereafter, we performed a chromatin immunoprecipitation (ChIP) assay to examine AR binding in these regions. We found that DHT treatment promoted AR recruitment to both AREs, but the enrichment was greater at the P1 site than the P2 site in both LNCaP and C4-2 cell lines. Moreover, we did not find any change in the enrichment of AR in cells treated with either low or high doses of DHT (Fig. 2, *E* and *F*). To further confirm the regulatory significance of AR binding to *MYB* promoter, we mutated the P1 site (5′-CAATGGTTCT-3′ to 5′-CAAGGCATAT-3′) in the promoter-reporter constructs. Our data indicate that PCa cells transfected with the mutant promoter-reporter plasmid failed to exhibit DHT-mediated luciferase activity induction. (Fig. 2, *G* and *H*).

### Repression of MYB expression by androgen occurs late due to its reduced mRNA stability

**Figure 3:**
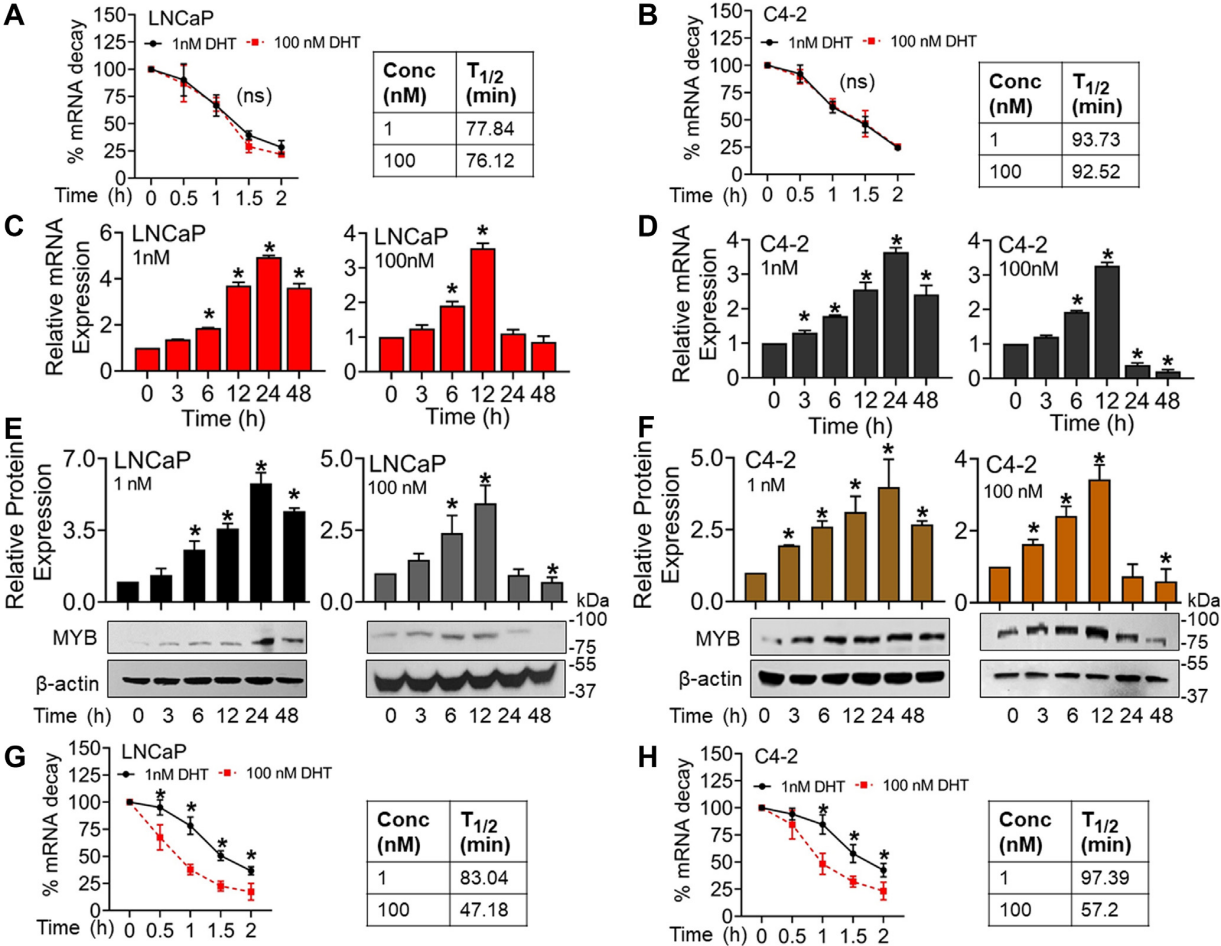
**MYB repression by high dose androgen occurs late due to the reduced stability of its mRNA.***A* and *B*, LNCaP and C4-2 cells were incubated with DHT (1 nM and 100 nM) for 6 h prior to the treatment with Actinomycin D. After 1 h, total RNA was isolated at different time intervals for up to 120 min, and MYB mRNA levels were analyzed by qRT PCR. 18s rRNA was used as an internal control. Percent mRNA decay was calculated from the earliest time point of analysis (0 min), and the data was presented as a line graph (mean ± S.D.). *C*–*F*, prostate cancer cells were treated with low (1 nM) or high (100 nM) doses of DHT for increasing time intervals (0–48 h). MYB expression was analyzed at both mRNA and protein levels by qRT-PCR and immunoblot assays, respectively. *ACTB* (qRT-PCR) and β-actin (immunoblot) were used as internal controls. Relative differences in MYB mRNA and protein levels are presented in the bar graph (mean ± S.D.), ∗*p* = < 0.05. *G* and *H*, LNCaP and C4-2 cells were incubated with DHT (1 nM and 100 nM) for 18 h prior to treatment with Actinomycin D. After 1 h, total RNA was isolated at different time points (0–120 min), and MYB mRNA expression was analyzed by qRT PCR. 18s rRNA was used as an internal control. Percent mRNA decay was calculated, and the data was presented as a line graph (mean ± S.D.). ∗*p* < 0.05; n.s. not significant. DHT, dihydrotestosterone.

Since we did not find differential transcriptional regulation of MYB by low and high-dose DHT treatment, we examined the possibility of a parallel posttranscriptional repressive mechanism. PCa cells were treated with low (1 nM) and high (100 nM) doses of DHT and, after 6 h, treated with actinomycin D to stop mRNA synthesis. After 1 h of actinomycin treatment, total RNA was isolated at different intervals (0–120 min), and MYB expression was analyzed. The data suggested no significant difference in MYB mRNA stability in cells treated with low and high doses of DHT (Fig. 3, *A* and *B*). Therefore, we next examined the time-dependent changes in MYB expression in PCa cells after the treatment with low and high doses of DHT. We observed that MYB expression increased at both mRNA and protein levels in DHT-treated cells at early time points, followed by a decline in expression at later time points. The decrease was, however, sharp and far greater in high-dose (100 nM) DHT-treated PCa cells and detected earlier (at 24 h) than the low-dose (1 nM) DHT-treated cells that showed a slight but significant decline at 48 h only at both RNA and protein levels (Fig. 3, *C*–*F*). Therefore, we assessed the changes in MYB mRNA stability at late time points (after 18 h) following the induction with DHT. The data revealed that the MYB mRNA half-life was decreased significantly in high-dose DHT-treated PCa cells (∼47.18 min for LNCaP and ∼57.2 min for C4-2) compared to the low-dose DHT-treated cells (∼83.04 min for LNCaP and ∼97.39 min for C4-2) (Fig. 3, *G* and *H*).

### Treatment of PCa cells with DHT induces miR-150 expression

**Figure 4:**
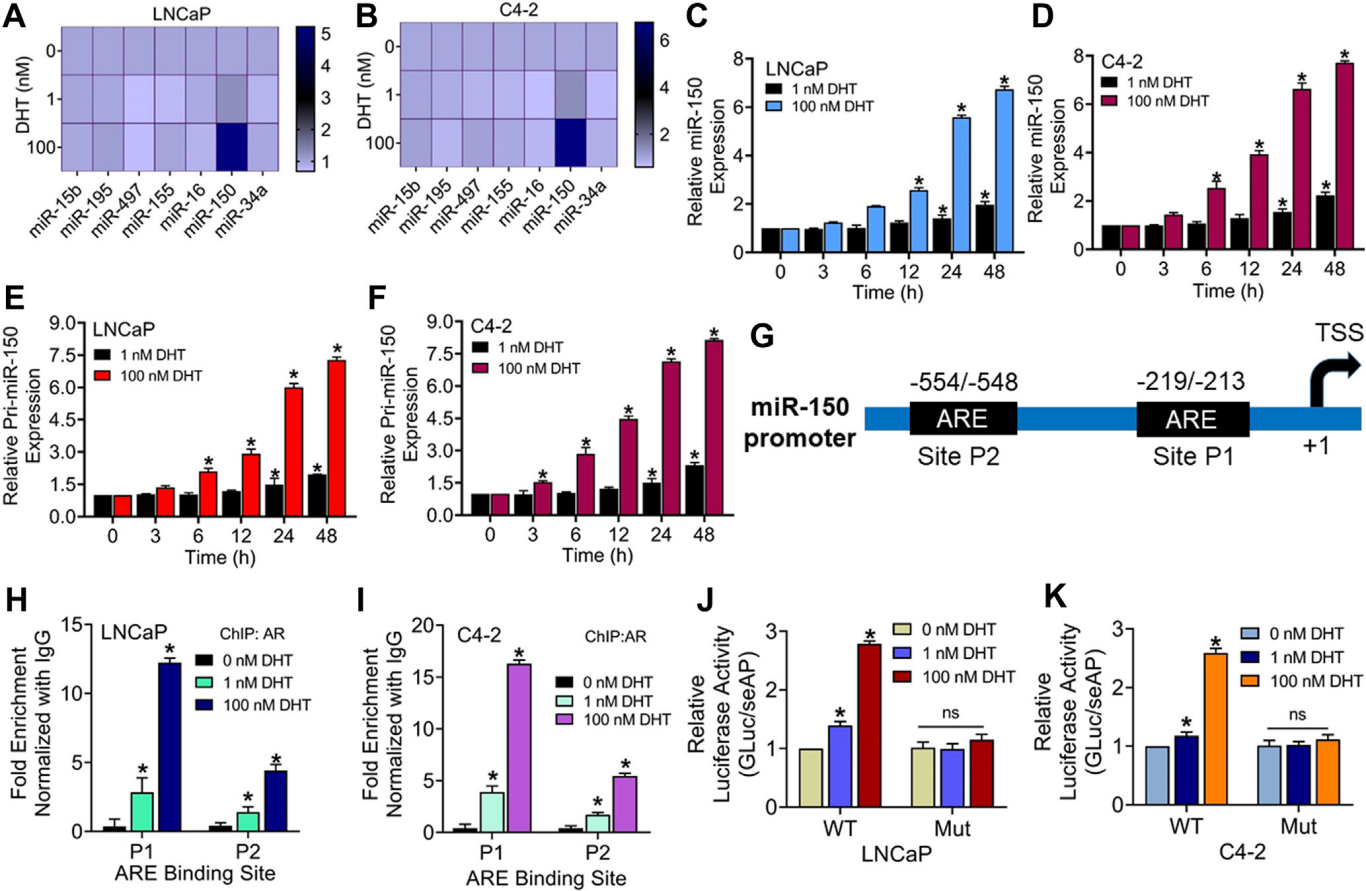
**High dose androgen treatment induces miR-150 expression in prostate cancer cells.***A* and *B*, LNCaP and C4-2 cells were treated with DHT (1.0 nM, 100 nM) for 24 h, and the levels of a set of MYB-targeting microRNAs were analyzed by qRT-PCR. U6 was used as an internal control. *C*–*F*, cells were treated with *low* (1 nM) and *high* DHT (100 nM) doses for increasing time intervals (0–48 h), and expression of miR-150 (*C* and *D*) or pri-miR-150 (*E* and *F*) was examined. *G*, *in silico* analysis of miR-150 promoter identified two AREs, P1 (-213 to -219) and P2 (-548 to -554), preceding the transcription start site. *H* and *I*, cells were treated with DHT (1.0 nM and 100 nM) for 24 h, and after that, ChIP assay was performed using AR-specific and control IgG antibodies, followed by qRT-PCR using primer sets flanking ARE. Fold enrichment (mean ± S D) from three replicates. *J* and *K*, LNCaP and C4-2 cells were transfected with WT or mutated (Mut) miR-150 promoter-reporter constructs for 24 h and then treated with either *low* (1 nM) or *high* (100 nM) DHT doses. After 24 h, luciferase activity was measured and normalized with seAP. Data was presented as relative fold change in a bar graph. ∗*p* = < 0.05, n.s. not significant. AR, androgen receptor; ARE, androgen-responsive element; ChIP, chromatin immunoprecipitation; DHT, dihydrotestosterone; seAP, secreted alkaline phosphatase.

miRNAs or miR play an important role in posttranscriptional gene repression and have been shown to mediate gene regulatory and phenotypic effects of the androgen signaling (23, 24). Therefore, we examined the possibility that they could mediate androgen-induced repression of MYB that we observed at late time points in DHT-treated PCa cells. Using target predictive web-based applications (miRDB and TargetScan), we selected a list of MYB-targeting miRNAs (miR-15b; miR-195; miR-497; miR-155; miR-16; miR-150; miR-34a) based on their target scores (Table S1). After that, we examined their expression in LNCaP and C4-2 cell lines following treatments with low (1 nM) and high (100 nM) doses of DHT. The results show an upregulation of miR-150 with a greater induction observed in high-dose DHT-treated LNCaP and C4-2 cells (Fig. 4, *A* and *B*). Further, in a time-course (0–48 h) experiment, we observed a steady increase in miR-150 expression, with a more profound upregulation observed in cells treated with high-dose DHT (Fig. 4, *C* and *D*). A similar expression pattern was also observed for a primary transcript of miR-150 (pri-miR-150), suggesting that differential expression of miR-150 in low- and high-dose DHT-treated PCa cells did not result from altered processing (Fig. 4, *E* and *F*). *In silico* analysis identified two putative AREs, P1 (-219/-213) and P2 (-554/-548), in the promoter (1000 bp regions upstream of miR-150 transcription start site) by using PROMO-ALGGEN and TFBIND (Fig. 4*G*). Further, in the ChIP assay, we observed significantly higher enrichment of AR in the proximal ARE in cells treated with high-dose DHT than those treated with low-dose DHT (Fig. 4, *H* and *I*). To confirm the regulatory significance of AR binding to miR-150 promoter, we mutated the P1 site (5′-TGCTGGTTCT-3′ to 5′-TGCTCCAAGT-3′) in the promoter-reporter plasmid. The data show that PCa cells transfected with WT miR-150 promoter-reporter construct exhibited significantly higher induction of luciferase activity when treated with high-dose DHT, compared to when they received low-dose DHT treatment. Furthermore, PCa cells transfected with mutant miR-150 promoter-reporter plasmid did not exhibit any significant induction of luciferase activity following treatment with either low or high dose of DHT (Fig. 4, *J* and *K*).

### Inhibition of miR-150 abrogates the repressive effect of high-dose DHT on MYB expression

**Figure 5:**
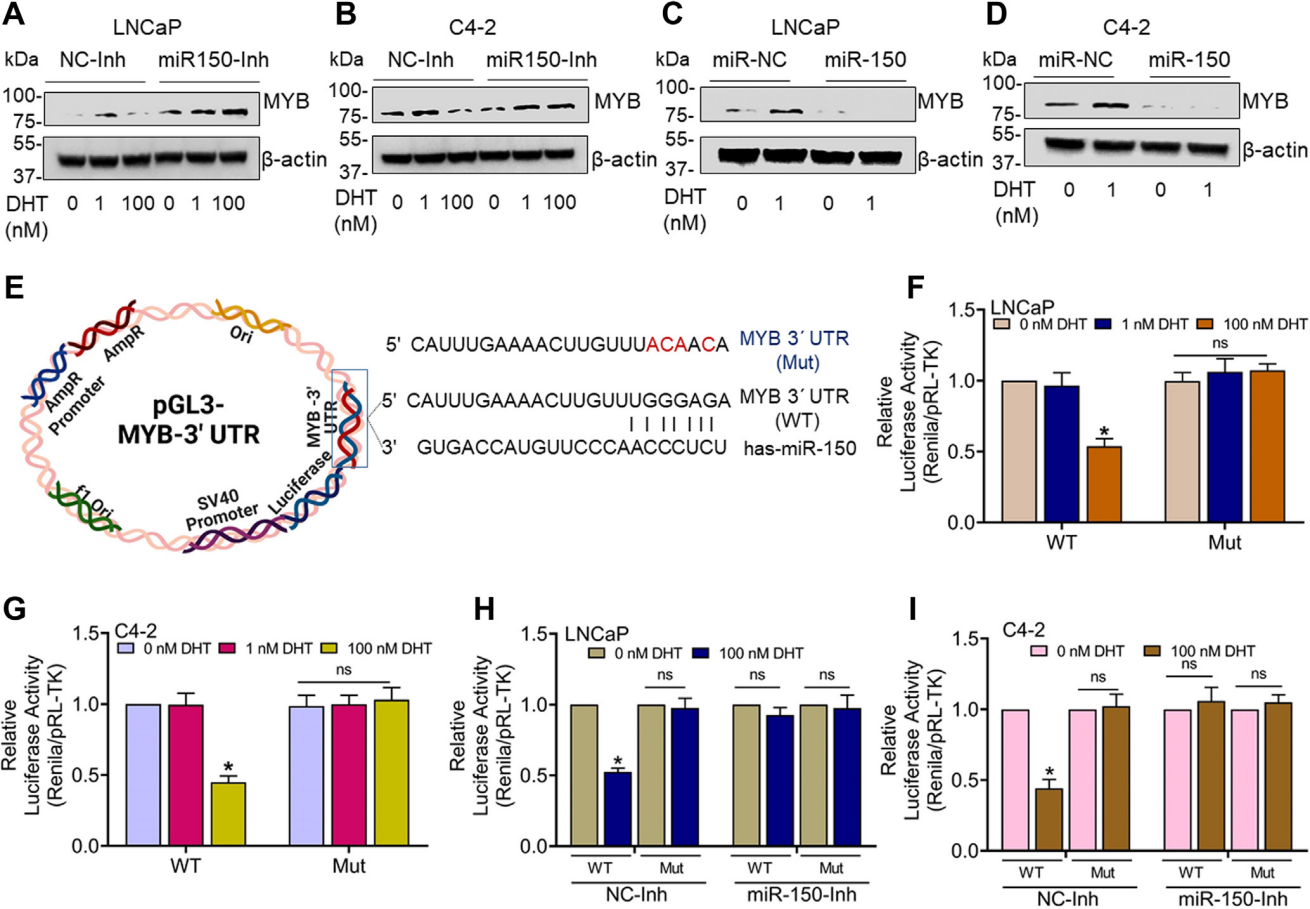
**Effect of miR-150 inhibition and restoration on MYB expression in low- and high-dose DHT-treated prostate cancer cells.***A* and *B*, LNCaP and C4-2 cells were transfected with negative control-inhibitor (NC-Inh) or miR-150 inhibitor (miR150-Inh) for 24 h, followed by low and high doses treatment of DHT for another 24 h. MYB expression was analyzed by Western blotting. β-actin was used as a loading control. *C* and *D*, cells were transfected with negative control miRNA mimic (miR-NC) or miR-150 mimic (miR-150) for 24 h, followed by DHT (1.0 nM) treatment for 24 h and MYB expression analysis. *E*–*G*, prostate cancer cells were transfected with a luciferase reporter plasmid carrying MYB-3ʹUTR-WT (WT) sequence containing a putative-binding site for miR-150 or MYB-3ʹUTR-Mut (Mutant) where miR-150 binding site is mutated. Cells were also cotransfected with Renilla luciferase plasmid (pRL/TK) to control transfection efficiency. After 24 h, cells received treatment with low and high doses of DHT, and luciferase activity was examined after 24 h of incubation. The data is presented as normalized luciferase activity (mean ± SD), ∗*p* = < 0.05. *H* and *I*, LNCaP and C4-2 cells were cotransfected with pGL3-MYB-3′UTR WT or pGL3-MYB-3′UTR mutant (Mut) luciferase along with either control-inhibitor (NC-Inh) or miR-150 inhibitor (miR150-Inh) for 24 h. Thereafter, cells were treated with low and high DHT doses for 24 h. Luciferase activity was examined, and the data presented as the normalized luciferase activity (mean ± SD), ∗*p* = < 0.05, n.s. not significant. DHT, dihydrotestosterone.

To examine the functional significance of DHT-induced miR-150, we treated LNCaP and C4-2 with anti–miR-150 or nontargeted control miRNA 24 h prior to DHT treatment. We observed that MYB repression caused by high-dose DHT treatment was completely abolished in cells pretreated with anti–miR-150 (Fig. 5, *A* and *B*). To further establish the role of miR-150 in MYB repression, we treated LNCaP and C4-2 cells with miR-150 mimics 24 h prior to treatment with low dose DHT (1.0 nM). We observed that the treatment with miR-150 mimics suppressed basal and low-dose DHT-induced MYB expression in both cell lines (Fig. 5, *C* and *D*). To further confirm MYB repression by miR-150 in PCa cells following DHT treatment, we performed a reporter assay using a plasmid that had MYB-3′UTR sequence downstream of luciferase cDNA containing miR-150 targeted sequence (Fig. 5*E*). In addition, we mutated miR-150 binding sequence in the MYB 3′-UTR (GTTTGGGAGA-3′ to 5′-GTTTACAACA-3′) and used this mutant plasmid to validate the regulatory significance of direct miR-150/MYB 3′UTR binding. PCa cells were transfected with WT or mutant MYB-3′UTR carrying reporter plasmids. Cells were also cotransfected with pRL-TK plasmid to control for transfection efficiency. Following 24 h of transfection, cells were treated with low and high doses of DHT for another 24 h. The data show a significant reduction in normalized luciferase activity in PCa cells in WT-MYB 3′UTR reporter plasmid when treated with high-dose DHT. However, no repression in luciferase activity was reported in cells transfected with mutant MYB-3′UTR reporter plasmid (Fig. 5, *F* and *G*). Moreover, transfection of PCa cells with anti–miR-150 24 h prior to high-dose DHT treatment abolished its repressive effect on the luciferase activity. No effect of miR-150 inhibitor was reported in mutant MYB -3′-UTR reporter–transfected PCa cells (Fig. 5, *H* and *I*). In contrast, transfection of PCa cells with miR-150 mimics reduced luciferase activity in both LNCaP and C4-2 cells, whereas no repression was observed in mutant MYB -3′-UTR reporter–transfected PCa cells (Fig. S4, *A* and *B*).

### Inhibition of MYB abrogates low-dose DHT-induced growth, whereas miR-150 inhibition leads to growth induction in high-dose DHT-treated PCa cells

**Figure 6:**
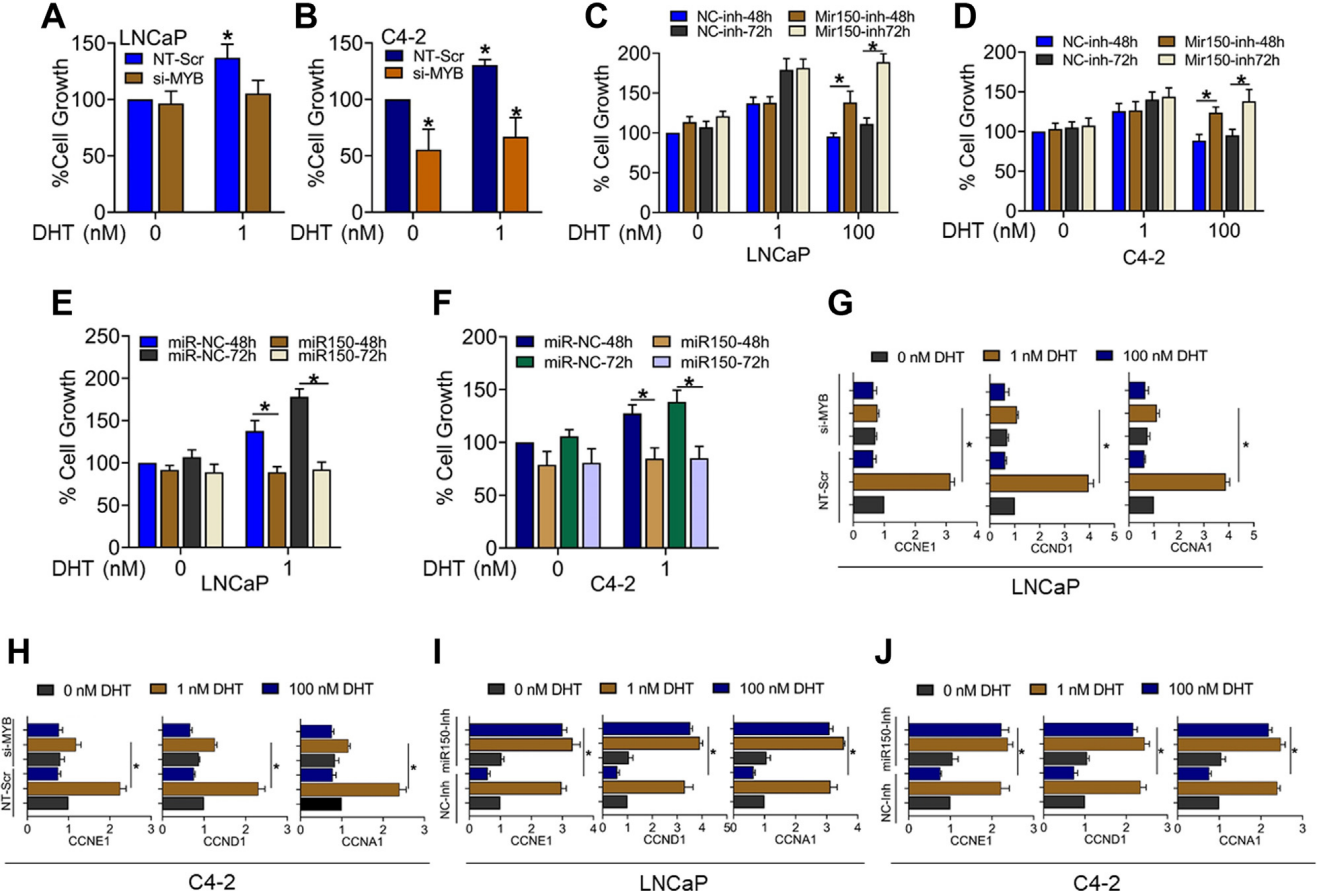
**Effect of direct and indirect inhibition of MYB on PCa cells growth and expression of cell cycle–associated genes.** LNCaP (*A*) and C4-2 cells (*B*) were transfected with nontargeted scramble (NT-Scr) or MYB- specific siRNA (si-MYB) for 24 h, followed by low and high DHT treatment for 72 h. After incubation, cell growth was measured by WST-1 assay. LNCaP and C4-2 cells were transfected with either miR-150 inhibitor (miR150-Inh) (*C* and *D*) or miR-150 mimic (*E* and *F*) along with their respective negative controls for 24 h, and then cells were exposed to low and high DHT for 48 and 72 h. After that, cell growth was measured by WST-1 assay, and data were presented as a bar graph. LNCaP and C4-2 cells were transfected with nontargeted scramble (NT-Scr) or MYB- specific siRNA (si-MYB) for 24 h, followed by low and high DHT treatment for an additional 24 h. Total RNA was collected, and cell cycle–specific gene expression (*CCNA1*, *CCNE1*, and *CCND1*) was analyzed by qRT-PCR (*G* and *H*). Similarly, the expression of *CCNA1*, *CCNE1*, and *CCND1* was also analyzed in miR-150 inhibitor–transfected LNCaP and C4-2 cells treated with a low or high dose of DHT. *ACTB* was used as an internal control (*I* and *J*). ∗*p* = < 0.05. DHT, dihydrotestosterone; PCa, prostate cancer.

To examine the significance of biphasic MYB regulation upon DHT treatment on prostate tumor cell growth, we silenced MYB expression by RNA interference prior to DHT treatment. LNCaP and C4-2 cells were transfected with nontargeted scrambled or MYB-targeted siRNAs for 24 h and then treated with DHT (1.0 nM), and cell growth was examined after 72 h. MYB silencing following siRNA treatment was confirmed by immunoblotting (Fig. S5). The data show a significant reduction in low DHT-mediated growth induction in both LNCaP and C4-2 cells (Fig. 6, *A* and *B*). In the next set of experiments, we treated the PCa cells with miR-150 inhibitor followed by high-dose (100 nM) DHT treatment and measured the effect on cell proliferation. Our data show that PCa cells transfected with anti–miR-150 were rescued from the growth repressive effect of high-dose DHT (Fig. 6, *C* and *D*). Furthermore, transfection of LNCaP and C4-2 cells with miR-150 mimics significantly reduced their growth following low-dose (1.0 nm) DHT treatment (Fig. 6, *E* and *F*). We also examined the effect of MYB silencing and miR-150 inhibition on the expression of cell cycle–associated MYB-regulated genes (*CCND1*, *CCNE1*, *CCNA1*) upon treatment with low and high doses of DHT. As expected, after the knockdown of MYB, the induction of cell cycle factors (*CCND1*, *CCNE1*, *CCNA1*) by a low dosage of DHT was completely blocked in both LNCaP and C4-2 cell lines (Fig. 6, *G* and *H*). Next, our data demonstrated that low doses of DHT upregulated the expression of *CCND1*, *CCNE1*, and *CCNA1* levels in both LNCaP and C4-2 that remained unaltered in the presence of anti–miR-150, while high DHT suppressed *CCND1*, *CCNE1*, and *CCNA1* in both LNCaP and C4-2. Furthermore, the expression of *CCND1*, *CCNE1*, and *CCNA1* was increased in anti–miR-150–transfected high DHT-treated PCa cells (Fig. 6, *I* and *J*).

## Discussion

In this study, we identified a novel mechanism underlying MYB dysregulation in PCa. We observed that androgen treatment regulated MYB expression in a biphasic dose-dependent manner paralleling its effect on the growth of PCa cells. MYB induction by androgen occurred early *via* a transcriptional mechanism involving AR binding to its promoter. In contrast, MYB repression, especially by high-dose androgen, occurred late *via* a miR-150–mediated posttranscriptional mechanism. Moreover, we observed that MYB induction by androgen was necessary for its growth-promoting effect, whereas its repression by exogenous miR-150 led to suppression of growth of PCa cells.

Androgen signaling plays an important role in PCa pathogenesis and remains a primary target for therapeutic intervention (7, 25). Aberrant reactivation of androgen signaling is also suggested to be a major mechanism for therapeutic failure following androgen-deprivation therapy (7, 26). In our recent study, we demonstrated that MYB functionally cooperated with AR to sustain its transcriptional activity under androgen-depleted condition and promoted castration resistance *in vivo* (22). These findings contradicted previous research that demonstrated a compensatory role of MYB in castration resistance (21). Interestingly, we also previously observed that MYB is aberrantly upregulated in PCa cell lines that lack AR expression (17). Therefore, our findings bring some clarity by demonstrating that androgen can, in fact, have both stimulatory and repressive effects on MYB expression. Moreover, we observed that androgen inhibited MYB expression at lower doses in CR C4-2 cells than the castration-sensitive LNCaP cells. It could be because of the fact that C4-2 cells express higher levels of AR than the LNCaP cells (22). Interestingly, C4-2 cells also retained a high level of MYB expression when cultured under androgen-deprived conditions suggesting that its aberrant overexpression in these cells involves additional androgen-independent mechanism(s).

A dose-dependent biphasic response of androgens has been reported for several genes (8, 9). Similarly, androgens are also shown to have both growth-promoting and growth-repressive effects on PCa cells as well (27, 28). In fact, high-dose androgen supplementation is being evaluated as a promising therapy for PCa in clinical trials (29). Early observations of the biphasic effect of androgen signaling prompted investigations to delineate the underlying molecular mechanisms (30). It was shown that the activity of the E2F-1 transcription factor, a major gatekeeper of the transition from the G1- to S-phase of the cell cycle, was differentially regulated by androgens (10). Repression of E2F1 transcriptional activity paralleled with hypophosphorylation of retinoblastoma protein, resulting in reduced expression of target mRNAs. This emerged as a major mechanism of the dose-dependent effect of androgen on PCa cells in a recent study. It was shown that high-dose androgen increased the recruitment of hypophosphorylated retinoblastoma protein on the promoters of DNA-replicative genes leading to suppression of AR function (31). Our finding on MYB is different from these observations since we find an inducing effect of androgen on the transcriptional activity of MYB promoter at both low and high doses. Our study revealed that MYB repression occurred late *via* an miRNA-mediated posttranscriptional mechanism and was more prominent in high-dose androgen-treated PCa cells. This is a novel observation and exposes a yet unidentified mode of dose-dependent differential actions of androgens in PCa.

Posttranscriptional regulation of MYB *via* miRNA-mediated mechanisms has been demonstrated in multiple cancer cell types and during the cellular differentiation (32, 33, 34). Also, several miRNAs have been identified to exhibit aberrant expression in PCa and play a role in pathobiology and therapy resistance (35, 36). A differential expression of miR-150 has also been reported in PCa with some conflicting data on its functional significance (24, 37, 38, 39). One study found that miR-150 was significantly downregulated in prostate tumor tissues compared to the adjacent normal tissue, and its expression was inversely associated with the survival of patients (37). miR-150 has also been shown to suppress EMT, invasion, and metastasis of PCa cells (38). In another study, decreased levels of miR-150 in the plasma of PCa patients were reported (39). In contrast, two studies reported the tumor-promoting function of miR-150 in PCa supporting cell proliferation, malignant behavior, and chemoresistance (40, 41). In these contexts, our observation of the dose- and time-dependent androgen-mediated differential regulation of miR-150 is intriguing and should be explored further. It is likely that androgen-mediated miR-150 upregulation acts as a feedback mechanism. Alternatively, miR-150 targets may also engage in functional crosstalk with androgen signaling to yield diverse effects on cancer cell phenotypes. This seems particularly true since we have earlier shown that MYB modulates AR transcriptional activity and its occupancy on gene promoters (22).

Another interesting finding in our study is differential regulation of miR-150 promoter activity in PCa cells treated with low and high doses of androgen. While we did not observe a differential induction of the MYB promoter, the transcriptional activity of the miR-150 promoter was dramatically high in cells treated with a high dose of DHT. It could probably result from varying affinity of AR to different genomic sequences within AREs (42, 43). In a parallel study, we have observed that PCa cells treated with high doses of DHT accumulate greater levels of AR in the nucleus than those treated with a low dose (unpublished data). It could be one reason why we see a greater induction of miR-150 in cells treated with high-dose DHT. These exciting findings provide additional clues for a differential action of androgen in PCa cells. Our observations also bring MYB and miR-150 to the forefront of prostate tumor biology by demonstrating their significance in the growth-stimulatory and growth-repressive effects of androgens. The role of MYB in the biology of prostate and other cancers has been demonstrated by us and others (17, 19, 22). Similarly, multiple other targets of miR-150 have also been characterized (44, 45, 46). Therefore, it will be interesting to expand upon our findings. Specially, the role of MYB and miR-150 should be explored in PCa cells that do not express a functional AR. This is significant since a recent study has shown decreased levels of miR-150 in serum extracellular vesicles CR PCa patients associated it with treatment-induced neuroendocrine differentiation (47).

In summary, our study identifies a novel androgen-mediated regulatory mechanism of MYB in PCa that appears to be a defining feature in its biphasic growth responses. We also establish miR-150 as an androgen-responsive miRNA, which may serve as a control mechanism for androgen action. These findings have important translational implications considering the fact that androgen signaling remains a major therapeutic target for PCa management.

## Experimental procedures

### Cell lines and culture conditions

LNCaP (ATCC) and C4-2 (UroCor) cell lines were maintained in Roswell Park Memorial Institute (RPMI) 1640 media (Invitrogen). LAPC4 and VCaP (ATCC) cells were maintained in Dulbecco’s modified Eagle’s media (Invitrogen). All culture media were supplemented with 10% fetal bovine serum (Atlanta Biologicals) and 1.0% penicillin and streptomycin each (Invitrogen). MYB-overexpressing (LNCaP-MYB), MYB-knockdown (C4-2-shMYB), and their respective control cell lines (LNCaP-Neo and C4-2-Scr) generated by us in a previous study (17) were maintained in antibiotic selection media. The media was replaced with antibiotic-free media prior to assays. All cell lines were authenticated intermittently using short-tandem repeats genotyping or expression of marker proteins. Cells were also routinely confirmed to be free from *mycoplasma* contamination using MycoAlert *Mycoplasma* Detection Kit (Lonza).

### Reagents and antibodies

RNA isolation kit was purchased from Qiagen. Nitrocellulose membrane, CSS, and Penicillin-Streptomycin antibiotics were purchased from Thermo Fisher Scientific. WST-1 reagent was purchased from BioVision, Inc. MYB promoter-reporter construct (GLuc-ON MYB; HPRM34981-PG02), MYB mutant promoter-reporter construct (GLuc-ON MYB; HPRM34981-PG02-01), has-miR-150 WT and mutant (CS-HPRM54623-PG04-01 and CS-HPRM54623-PG04-02) were purchased from GeneCopoeia. p-GL3-MYB-3′UTR (Cat. # 25798) (Firefly Luciferase) (48) was purchased from Addgene, p-GL3-MYB-3′ UTR-mutant (VB20819-12933men) was purchased from VectorBuilder, and pRL-Tk (Renilla Luciferase) plasmid constructs was purchased from Promega. X-tremeGENE HP DNA Transfection Reagent, X-tremeGENE siRNA transfection Reagent, DHT, and actinomycin D were obtained from Sigma-Aldrich. Antibodies against MYB (Cat. # 59995S), AR (Cat. # 5153S), and horseradish peroxidase (HRP)-β-actin (Cat. # 12620S) were purchased from Cell Signaling Technology. Anti-rabbit HRP-conjugated secondary antibodies (Cat. # NB7160) were obtained from Novus biological (Centennial, CO). A dual luciferase kit was purchased from Promega. MYB- (Cat.# L-003910-00-0005) and AR- (Cat.# L-003400-00-0005) specific siRNAs were purchased from Dharmacon Horizon Discovery, and hsa-miR-150-5p miRNA Precursor (Cat.# 4464066) (miR-150), hsa-miR-150-5p miRNA inhibitor (Cat.# 4464084) (miR-150-Inh), Pre-miR miRNA Precursor Negative Control #1 (Cat.# AM17110), Anti-miR miRNA Inhibitor Negative Control #1 (Cat.# AM17010), and customize gene-specific primer pairs (Table S2) were purchased from Thermo Fisher Scientific.

### RNA isolation and quantitative real-time RT-PCR

Total RNA was isolated from cells using a Qiagen RNeasy purification kit and quantified using Nanodrop-1000 (Thermo Fisher Scientific). Two micrograms of total RNA were used for cDNA synthesis using the High-Capacity Complementary DNA Reverse Transcription Kit (Applied Biosystems). Quantitative RT-PCR (qRT-PCR) was performed in 96-well PCR plates by using SYBRGreen Master Mix (Applied Biosystems) with gene-specific primer pairs (Table S2) on an iCycler system (Bio-Rad Laboratories). TaqMan Pri-miRNA kit (Thermo Fisher Scientific) was used to detect pri-mir-150 and RNU6B levels. The thermal conditions for real-time PCR were as follows: step 1: 95 °C for 10 min, step 2: 95 °C for 10 s, followed by 55 to 60 °C for 45 s (×40 cycles).

### Protein extraction and immunoblotting

Cells were harvested, and total protein was collected as described previously (22, 49). Briefly, cells were lysed with NP-40/RIPA [150 mM NaCl, 50 mM Tris pH 8.0, 1% NP-40, 0.5% deoxycholate, and 0.1% SDS] buffer containing protease and phosphatase inhibitors (Thermo Fisher Scientific). A 20 to 60 μg of total protein was resolved on 10% PAGE and transferred onto PVDF membranes (Millipore Sigma). Next, membranes were incubated with MYB or AR-specific primary antibodies followed by HRP-labeled secondary antibodies. The signal was generated using SuperSignal West Femto Maximum Sensitivity Substrate Kit (Thermo Fisher Scientific). Bands were captured using the ChemiDoc XRS+ system (Bio-Rad Laboratories).

### Cell growth assay

The cell growth assay was performed as described previously (50). Briefly, cells (1 × 10^4^/well) were seeded in 96-well plates for 24 h. Thereafter, media was replaced by CSS containing fresh media and allowed to grow for 24 h. Cells were then treated with different concentrations (0–100 nM) of DHT for different time intervals (48–96 h), and cell growth was measured using WST-1 assay.

### ChIP assay

The assay was performed using ChIP-IT Express Enzymatic Kit (Active Motif) as described earlier (22). In brief, cells (5 × 10^6^) were cultured in 150 mm dishes to 60 to 70% confluence and treated with different concentrations of DHT for 24 h. Thereafter, cells were incubated with 1.0% formaldehyde (Sigma-Aldrich) for 10 min to cross-link DNA and proteins and scraped in an ice-cold cell-scraping solution supplemented with protease inhibitors. The cross-linked chromatin (DNA–protein complexes) was enzymatically sheared using ChIP-IT Express Enzymatic Kit (Active Motif) and subjected to immunoprecipitation using protein G magnetic beads using anti-AR or normal rabbit IgG (control) antibodies. Magnetic beads were washed and cross-linking reversed using reverse cross-linking buffer followed by proteinase K digestion and DNA purification. The immunoprecipitated DNA was purified and subjected to qRT-PCR using specific primer sets as mentioned in Table S2.

### Luciferase reporter assay

For the assay of has-miR-150 promoter activity, cells were transfected with a dual promoter reporter construct containing Gaussia Luciferase (GLuc) downstream of miR-150 promoter and seAP under constitutive CMV promoter using X-tremeGENE HP DNA Transfection Reagent as per manufacturer’s instructions. For the assay of MYB promoter activity, cells were cotransfected with promoter-reporter construct containing Gaussia Luciferase (GLuc) and seAP under constitutive CMV promoter. To assay the direct effect of miR-150 in posttranscriptional MYB regulation, cells were cotransfected with p-GL3-MYB-3′UTR (Firefly Luciferase) and pRL-Tk (Renilla Luciferase) plasmid constructs. After 24 h, media was replaced with fresh CSS media, followed by DHT treatment for 24 h. GLuc/SeAP activity was monitored in the cell supernatant by using Secrete-Pair Dual Luminescence Assay Kit (GeneCopoeia). Renilla Luciferase and Firefly Luciferase activities were measured in cell lysates using the Dual-Luciferase Reporter Assay System (Promega). SeAP and Renilla Luciferase activities were used to normalize the transfection efficiency.

### RNA stability assay

The stability of mRNA was measured using a modified method described elsewhere (51). Briefly, cells were grown to 60 to 80% confluence. Following 24 h of incubation in CSS media, cells were treated with DHT (1 nM or 100 nM) for either 6 h or 18 h. Subsequently, cells were treated with actinomycin D (10 μg/ml), and total RNA was isolated after a gap of 1 h at different time intervals (0–2 h). MYB mRNA levels were measured by qRT-PCR. 18s rRNA was used as an internal control.

### Statistical analysis

Statistical significance was determined by one-way ANOVA for multiple groups and unpaired Student's *t* test to analyze statistical significance between the two groups. A *p*-value of *p* <0.05 was considered statistically significant.

## Data availability

The data presented in this article are stored with us and available for sharing upon request.

## Supporting information

This article contains supporting information.

## Conflict of interest

The authors declare no conflict of interest.
