# Supplementary material for: Biphasic transcriptional and posttranscriptional regulation of MYB by androgen signaling mediates its growth control in prostate cancer
Source: J Biol Chem. 2022 Nov 19;299(1):102725. doi: 10.1016/j.jbc.2022.102725 (PMC9791434; doi:10.1016/j.jbc.2022.102725)
Supplement: Supporting information [file mmc1.docx]

**Biphasic transcriptional and post-transcriptional regulation of MYB by androgen signaling mediates its growth control in prostate cancer**

Srijan Acharya^1,2^, Shashi Anand^1,2^, Mohammad Aslam Khan^1,2^, Haseeb Zubair^1,2^, Sanjeev Kumar Srivastava^1,2^, Seema Singh^1,2,3^, and Ajay Pratap Singh^1,2,3^*

*^1^Department of Pathology, College of Medicine, University of South Alabama, Mobile, AL 36617; ^2^Cancer Biology Program, Mitchell Cancer Institute, University of South Alabama, Mobile, AL 36604; ^3^Department of Biochemistry and Molecular Biology, College of Medicine, University of South Alabama, Mobile, AL 36688;*

*****Correspondence to:

Ajay Pratap Singh, PhD

Department of Pathology, College of Medicine

Mitchell Cancer Institute, University of South Alabama

1660 Springhill Avenue, Mobile, AL 36604

Tel: +1 251-445-9843, Fax: +1 251-460-6994

Email: asingh@southalabama.edu

**Supplementary Figures**

**Supplementary Fig. S1**

**
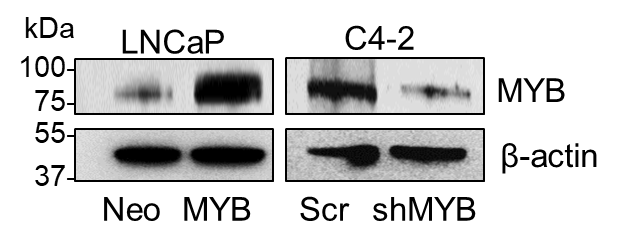
**

**Figure S1. MYB expression in genetically-engineered prostate cancer cell lines.** Total protein was isolated from MYB overexpressing (LNCaP-MYB) and MYB-silenced (C4-2-shMYB) stable cell lines along with their respective controls (LNCaP-Neo and C4-2-Scr). Equal amount of proteins from each cell lines was subjected to immunoblotting to examine MYB expression. β-actin was used as loading control.

**Supplementary Fig. S2**

**
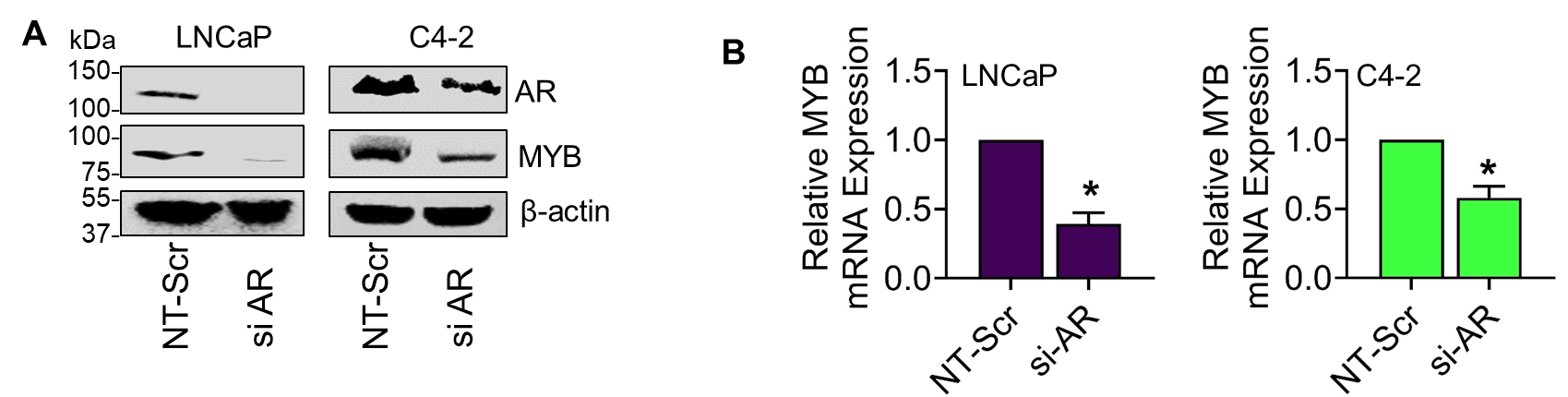
**

**Figure S2. Silencing of AR inhibits MYB expression at both mRNA and protein levels.** LNCaP and C4-2 cells were transfected with non-targeted scramble RNA (NT-Scr) or AR-specific siRNA (si-AR) for 24 h. Total protein was extracted and the expression of MYB and AR was examined using immunoblotting (**A**). Total RNA was isolated and the expression of MYB was analyzed using qRT-PCR (**B**). β-actin (immunoblot) and *ACTB* (qRT-PCR) were used as internal controls. **p < 0.05*.


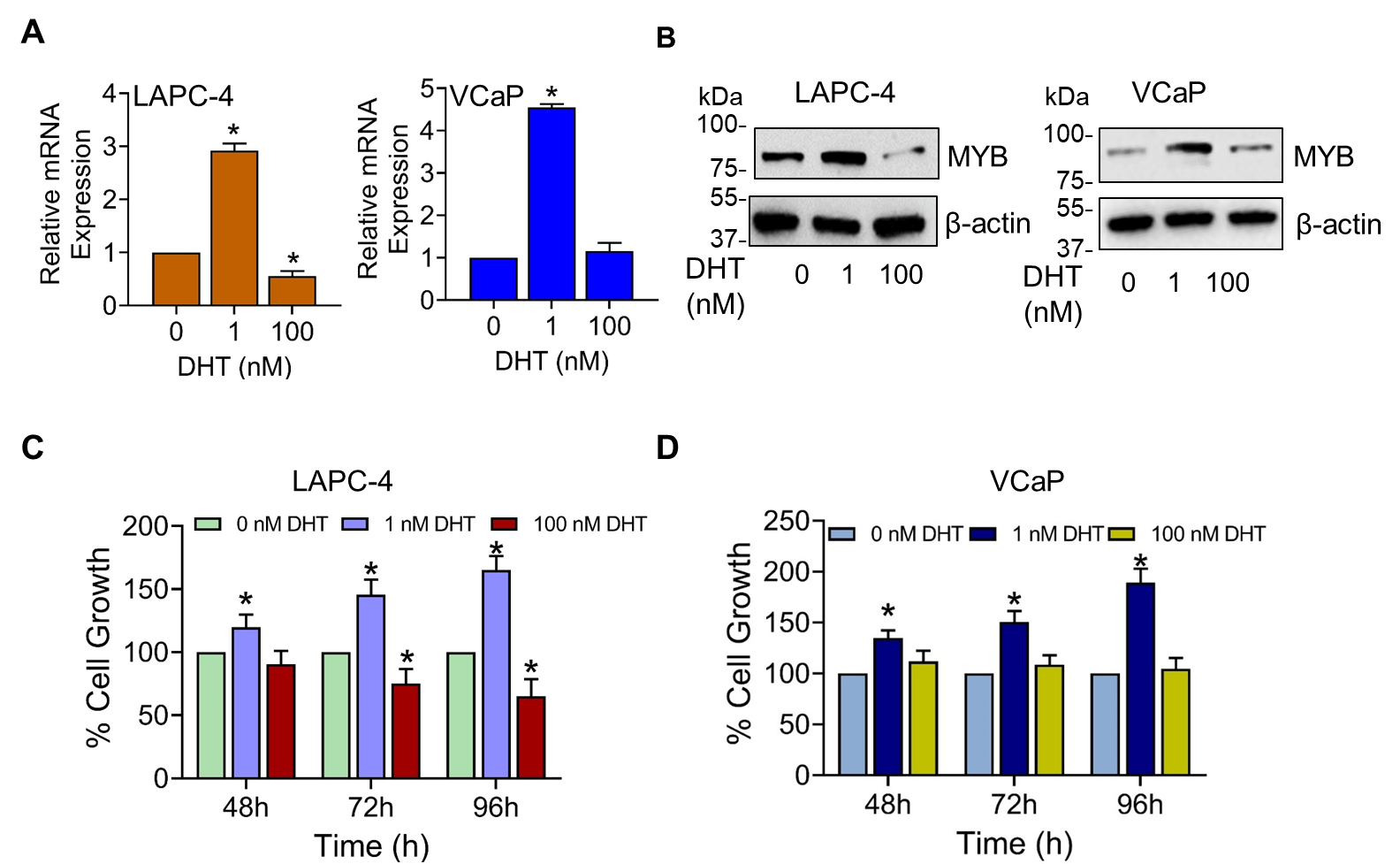
**Supplementary Fig. S3**

**Figure S3. MYB expression and cell growth in high and low dose DHT-treated LAPC-4 and VCaP cells.** were treated with low (1 nM) and high (100 nM) concentrations of DHT for 48 h in CSS-containing media, and MYB expression was examined at mRNA (**A**) and protein (**B**) levels. *ACTB* (qRT-PCR) and β-actin (immunoblot) were used as internal controls. (**C, D**) To examine the effect of DHT on the growth of LAPC-4 and VCaP cells, both the cells were treated with low (1 nM) and high (100 nM) doses of DHT in CSS supplemented media and growth was measured at different time intervals (48-96 h). The data is presented as mean ± S.D; *p* < 0.05.

**Supplementary Fig. S4**

**
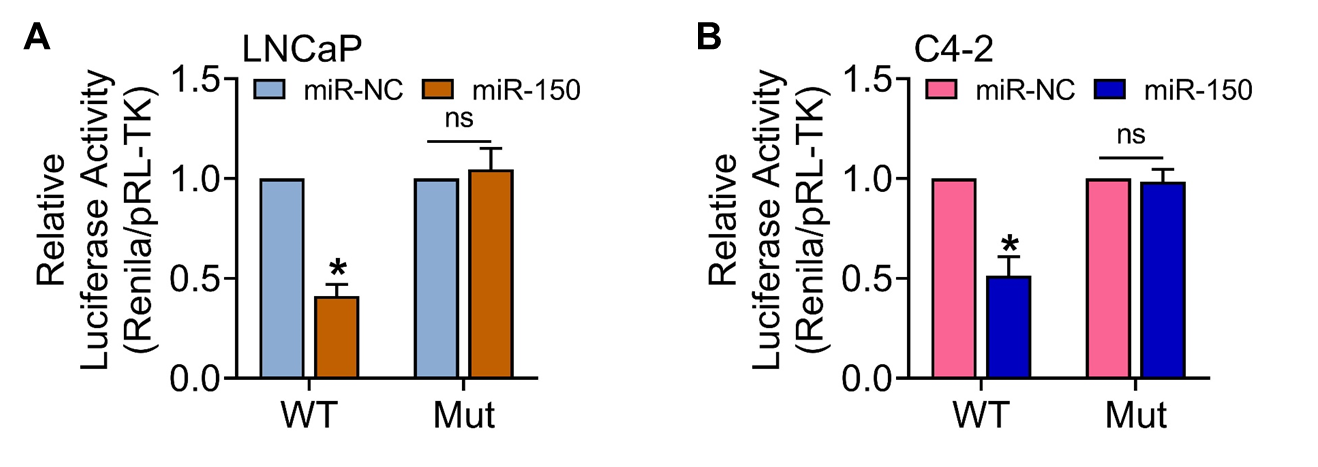
**

**Figure S4. miR-150 suppresses luciferase activity of MYB-3ʹUTR.**  (**A** and **B**) LNCaP and C4-2 cells were co-transfected with pGL3-MYB-3'UTR wild type (WT) or pGL3-MYB-3'UTR mutant (Mut) reporter and pRL-TK plasmids along with either miR-NC or miR-150 mimic for 24 h. Luciferase activity was measured in the cell lysates and data presented as relative luciferase activity normalized with Renilla luciferase. **p < 0.05*, n.s. not significant.

**Supplementary Fig. S5**

**
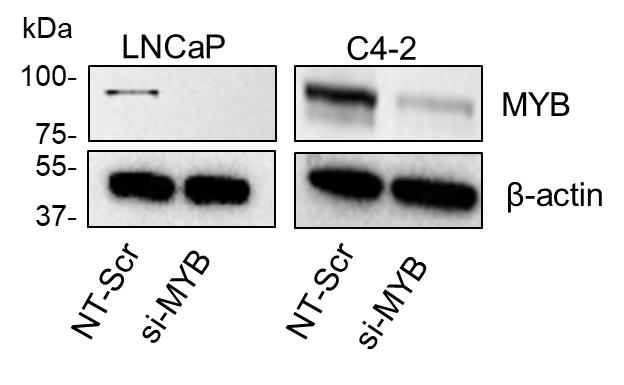
**

**Figure S5. siRNA-mediated MYB silencing in prostate cancer cells.** LNCaP and C4-2 cells were transfected with non-targeted scramble RNA (NT-Scr) or MYB-specific siRNA (si-MYB) for 24 h and then MYB expression was analyzed in the transfected cells by western blotting. Here β-actin was used as internal control.

**Supplementary Tables**


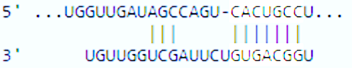
**Table S1:** List of miRNAs targeting MYB as analyzed from miRDB (http://www.mirdb.org/) and Target Scan (https://www.targetscan.org/vert_80/).

| **No.** | **miRNA** | **Predicted binding of MYB (top) and miRNA (bottom)** | **miRDB**  **(Target score)** | **Target Scan**  **(Targeting efficacy; context ++ score)** |
| --- | --- | --- | --- | --- |
| 1. | miR-15b | 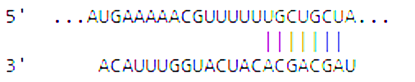 | 98 | 99 |
| 2. | miR-195 | 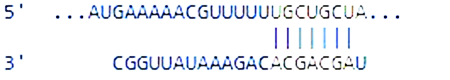 | 98 | 99 |
| 3. | miR-497 | 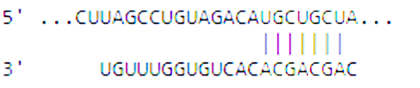 | 97 | 99 |
| 4. | miR-155 | 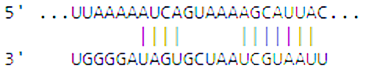 | 93 | 90 |
| 5. | miR-16 | 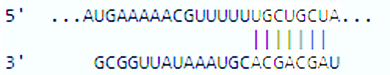 | 98 | 99 |
| 6. | miR-150 | 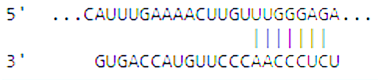 | 100 | 99 |
| 7. | miR-34a |  | 74 | 78 |

**Table S2:** List of primers used in this study

| **No.** | **Primer name** | **Sequence (5’-3’)** |
| --- | --- | --- |
| 1 | MYB | Forward- GAAAGCGTCACTTGGGGAAAA  Reverse- TGTTCGATTCGGGAGATAATTGG |
| 2 | AR | Forward- CCAGGGACCATGTTTTGCC  Reverse- CGAAGACGACAAGATGGACAA |
| 3 | β-Actin | Forward- CATGTACGTTGCTATCCAGGC  Reverse- CTCCTTAATGTCACGCACGAT |
| 4 | U6 | RT- GTCGTATCCAGTGCAGGGTCCGAGGT  Forward- CTCGCTTCGGCAGCACATATACT  Reverse- ACGCTTCACGAATTTGCGTGTC |
| 5 | miR-15b | RT- GTCGTATCCAGTGCAGGGTCCGAGGTATTCGCACTGGATACGACTGTAAA  Forward- TCGGCGTAGCAGCACATCATGG |
| 6 | miR-195 | RT- GTCGTATCCAGTGCAGGGTCCGAGGTATTCGCACTGGATACGACTAACCG  Forward- TCGGCGTAGCAGCACAGAAA |
| 7 | miR-497 | RT- GTCGTATCCAGTGCAGGGTCCGAGGTATTCGCACTGGATACGACCAAACA  Forward- TCGGCGCAGCAGCACACTGTGG |
| 8 | miR-155 | RT- GTCGTATCCAGTGCAGGGTCCGAGGTATTCGCACTGGATACGACAACCCC  Forward- TCGGCGTTAATGCTAATCGTGATA |
| 9 | miR-16 | RT- GTCGTATCCAGTGCAGGGTCCGAGGTATTCGCACTGGATACGACAACCGC  Forward- TCGGCGTAGCAGCACGTAAAT |
| 10 | miR-150 | RT- GTCGTATCCAGTGCAGGGTCCGAGGTATTCGCACTGGATACGACGGTCAC  Forward- TCGGCGTCTCCCAACCCTT |
| 11 | miR-34a | RT- GTCGTATCCAGTGCAGGGTCCGAGGTATTCGCACTGGATACGACAACCCC  Forward- TCGGCGTGGCAGTGTCTTAGCT |
| 12 | Universal | RT- AAAATATGGAACGCTTCACGAATTTG  Reverse- GTCGTATCCAGTGCAGGGTCCGAGGT |
| 13 | 18s RNA | Forward- CGGCGACGACCCATTCGAAC  Reverse- GAATCGAACCCTGATTCCCCGTC |
| 14 | MYB-P1 | Forward- GGGATACCGACCTCCAAAA  Reverse- GCTCCCCTCCCTGCACAGAGCGCGC |
| 15 | MYB-P2 | Forward- GGGACACCCACAAACCCAG  Reverse- GGGGGGATTTTAGGGGAGTTTT |
| 16 | miR150-P1 | Forward- GAACCAAGATCGCGCCATTGC  Reverse- GGGGAAGGACCTCTGCTGGGCC |
| 17 | miR150-P2 | Forward- CCTCCTATTCCCCTCTGGG  Reverse- GGGAGGGCGGGGGTTCCTGCC |
